# OGR1/GPR68 Modulates the Severity of Experimental Autoimmune Encephalomyelitis and Regulates Nitric Oxide Production by Macrophages

**DOI:** 10.1371/journal.pone.0148439

**Published:** 2016-02-01

**Authors:** Cheryl A. D’Souza, Fei Linda Zhao, Xujian Li, Yan Xu, Shannon E. Dunn, Li Zhang

**Affiliations:** 1 Toronto General Research Institute, University Health Network, Toronto, Ontario, Canada; 2 Department of Immunology, University of Toronto, Toronto, Ontario, Canada; 3 Department of Obstetrics and Gynecology, Indiana University School of Medicine, Indianapolis, Indiana, United States of America; 4 Women’s College Research Institute, Toronto, Ontario, Canada; 5 Department of Laboratory Medicine and Pathobiology and Immunology, University of Toronto, Toronto, Ontario, Canada; University Hospital of Heidelberg, GERMANY

## Abstract

Ovarian cancer G protein-coupled receptor 1 (OGR1) is a proton-sensing molecule that can detect decreases in extracellular pH that occur during inflammation. Although OGR1 has been shown to have pro-inflammatory functions in various diseases, its role in autoimmunity has not been examined. We therefore sought to determine whether OGR1 has a role in the development of T cell autoimmunity by contrasting the development of experimental autoimmune encephalomyelitis between wild type and OGR1-knockout mice. OGR1-knockout mice showed a drastically attenuated clinical course of disease that was associated with a profound reduction in the expansion of myelin oligodendrocyte glycoprotein 35-55-reactive T helper 1 (Th1) and Th17 cells in the periphery and a reduced accumulation of Th1 and Th17 effectors in the central nervous system. We determined that these impaired T cell responses in OGR1-knockout mice associated with a reduced frequency and number of dendritic cells in draining lymph nodes during EAE and a higher production of nitric oxide by macrophages. Our studies suggest that OGR1 plays a key role in regulating T cell responses during autoimmunity.

## Introduction

Multiple Sclerosis (MS) is a chronic inflammatory and demyelinating disease of the central nervous system (CNS) and is the most common neurological disorder affecting young adults [1]. It is generally thought that the incident attack of MS occurs when an unknown environmental agent triggers the activation and T helper 1 (Th1) and Th17 differentiation of myelin-reactive T cells in peripheral lymphoid organs. Upon trafficking to the CNS, pathogenic Th1 and Th17 cells secrete pro-inflammatory cytokines and chemokines that activate resident microglia and recruit other immune cells into the CNS. Together, immune cells and the cytotoxic factors secreted by these cells (i.e., TNF, nitric oxide, reactive oxygen species, glutamate, etc.) damage oligodendrocytes and axons, which leads to neurological disability [1]. Experimental autoimmune encephalomyelitis (EAE) is the common animal model of MS that recapitulates many immune features of the human disease, and is considered to be useful for modeling factors that regulate the initiation of autoimmunity [2–4].

One of the metabolic consequences of the development of autoimmune inflammation is acidification of the extracellular environment [5, 6]. Decreases in extracellular pH occur under a variety of inflammatory states, largely as a result of increased glycolytic activity and lactate production by immune cells [7]. For instance, during EAE, extracellular pH decreases from 7.4 to 6.6 in the inflamed spinal cord [5]. In rheumatoid arthritis, more modest decreases in pH (to 7.0–7.4) occur in the synovial fluid [6, 8], which correlate inversely with patient disease activity score [6]. Recent evidence suggests that ovarian cancer G protein-coupled receptor 1 (OGR1/GPR68) and other members of the OGR1-family of G protein-coupled receptors (GPCRs) are sensors of the mild decreases in extracellular pH that occur under inflammatory conditions [9]. These GPCRs have a set-point of regulation in the physiological range of pH (fully open between pH = 6.0–6.8 and fully closed at pH = 7.8) [9, 10] and sense changes in proton concentration through histidine residues in their extracellular domains [11]. Upon activation, OGR1 family members mediate pH-induced effects in cells through G protein-coupled increases in IP3/Ca^2+^or cAMP [9–12].

Recent emerging evidence suggests that OGR1 is expressed by various immune cells including macrophages [13–15], dendritic cells [16], T cells [17], and neutrophils [18] and has a pro-inflammatory function in various disease states [13, 16]. For example, OGR1-deficient mice (herein referred to as OGR1-KO mice) exhibit less severe airway inflammation in the ovalbumin (OVA)-induced sensitization/challenge model of asthma [16]. It has been also reported that syngeneic melanoma and prostate cell lines do not grow or engraft as well in OGR1-KO mice as a result of the higher tumoricidal activity of macrophages [17]. Furthermore, decreases in pH have been shown to trigger the OGR1-dependent increase in IL-6 secretion by human airway smooth muscle cells *in vitro* [19]. Despite these described immune functions, the role of OGR1 in autoimmunity has not yet been examined.

The objectives of this study were to determine the role of OGR1 in regulating the development of autoimmunity and the underlying mechanisms in the EAE model of MS. We observed that OGR1 deficiency led to a markedly attenuated clinical course of EAE that associated with a profound decrease in the expansion of myelin oligodendrocyte glycoprotein peptide 35–55 (MOG_35-55_)-reactive CD4^+^ T cells in the periphery and a reduced accumulation of Th1 and Th17 effector cells in the CNS. Furthermore, we pinpointed that these impaired T cell responses associated with a higher production of nitric oxide (NO) by macrophages and a reduced frequency of dendritic cells (DC) and macrophages in inflamed lymph nodes. Our results indicate that OGR1 has a key role in supporting the expansion of T cells during autoimmunity, providing the proof of concept that this molecule could be a novel target for therapeutic intervention in T cell-mediated autoimmune diseases such as MS.

## Materials and Methods

### Ethics Statement

All mice were housed in specific pathogen-free conditions in the Toronto Medical Discovery Tower animal facility. All experiments were performed under the Animal Use Protocol (#1125) that was approved by the University Health Network Animal Care Committee. Animals were euthanized by cervical dislocation that was carried out by trained personnel. Animal care was conducted in accordance with the policies and guidelines of the Canadian Council on Animal Care.

### Mice

Female wild type (WT) and 2D2 MOG T cell receptor transgenic mice on the C57BL/6J background were from the Jackson Laboratory. Breeders of OGR1-KO mice (C57BL/6J background) were kindly provided by Dr. L. Yan (Indiana University School of Medicine). All mice were housed in specific pathogen-free conditions at the Toronto Medical Discovery Tower animal facility (Toronto, Canada). All experiments were performed on female mice aged 8–12 weeks under the Animal Use Protocol (#1125) approved by the University Health Network Animal Care Committee.

### Induction of EAE and Clinical Evaluation

EAE was induced by subcutaneous injection of 100 μg MOG_35-55_ peptide (Prospec) emulsified with Complete Freund’s Adjuvant (CFA, 200 μg mycobacterium), followed by intravenous injection of 100 ng pertussis toxin (List Biological Laboratories, Inc.) on day 0 and day 2 post-immunization. CFA (4 mg/mL) was prepared by mixing Incomplete Freund’s Adjuvant (Difco Laboratories) with heat-killed *Mycobacterium Tuberculosis* H37Ra (Difco Laboratories). Mice were observed daily for clinical signs that were scored as follows: 0, no signs; 1, limp tail; 2, hind limb weakness; 3, complete hind limb paralysis on one or both sides; 4, hind limb paralysis and forelimb weakness; 5, moribund or dead. Mice with a score of 3 or above were administered saline subcutaneously (1 mL/day) and were provided Napa nectar in a petri dish on the bottom of the cage. In addition, their bladder was manually evacuated by gently applying pressure to the bladder. If mice obtained a score of 4, but did not have sufficient mobility to move towards a food source, they were examined by a clinical veterinarian and were monitored closely over a 24 h period. If the condition of the mouse did not improve after 24 h, the mouse was euthanized and was provided a score of 5 for the remainder of the study.

### Histological Assessment

Brain and spinal cord sections were collected from WT and OGR1-KO mice with EAE, fixed in 10% formalin, and embedded in paraffin for histological staining. Sections were stained with hematoxylin and eosin (H&E) and Luxol Fast Blue. Stained transverse sections of the spinal cord (N = 10−12/mouse) were scored blindly for the presence of meningitis, perivascular cuffs and demyelination as described previously [20].

### Quantification of CNS Effector CD4^+^ T cells

Mice were induced for EAE and were sacrificed on day 28 post-immunization. Brain and spinal cords were collected and CNS mononuclear cells were isolated by Percoll gradient centrifugation as previously described [21]. Cells were counted and were re-stimulated *in vitro* in complete RPMI-1640 medium (containing 10% FCS) with 50 ng/mL PMA and 500 ng/mL ionomycin in the presence of brefeldin A for 4 hours. These cells were then stained with antibodies specific for CD4, IFN-γ and IL-17 and were analyzed by flow cytometry (see details of antibodies and flow cytometry below).

### Measurement of Recall T Cell Response Against MOG_35-55_

Draining lymph nodes were harvested from mice on day 10 post-immunization and passed through 40 μM nylon cell strainers (BD Falcon) to obtain single-cell suspensions. Red blood cells were lysed by incubation in Lysis Buffer (0.14 M ammonium chloride, 0.02 M Tris, pH = 7.2) for 2 minutes and cells were counted using an automatic Vi-Cell cell counter (Beckman Coulter). Cells were labeled with 0.5 μM CFSE according to product directions (Life Technologies), were resuspended in complete RPMI-1640 medium and were cultured in 96 well plates (0.5 x 10^6^/well) together with 1–10 μg/mL MOG_35-55_ peptide for 3 days. Cells were then stained with viability dye and anti-CD4 antibody and flow cytometry was performed to determine the percent of CD4^+^ T cells that had divided. The levels of IFN-γ and IL-17 were measured in culture supernatants using ELISA kits (BioLegend).

### Measurement of T Cell Proliferation and Differentiation

CD4^+^ T cells were isolated from spleen and lymph nodes of mice by negative selection using the MACs separation system (Miltenyi Biotec). Cells were resuspended in complete RPMI and were plated with soluble anti-mouse CD28 antibody (2 μg/mL) on 96-well plates that were pre-coated with LEAF purified anti-mouse CD3 antibody (2 μg/mL) (both from BioLegend). For proliferation assays, cells were first labeled with 0.5 μM CFSE prior to culture. For Th differentiation assays, cells were stimulated without additional reagents (Th0 conditions), in the presence of 10 μg/mL anti-IL-4 (BioLegend) and 5 ng/mL recombinant mouse IL-12 (Peprotech) (Th1 conditions), or with 10 μg/mL anti-IL-4, 10 μg/mL anti-IFN-γ (BioLegend), 20 ng/mL recombinant mouse IL-6 (Peprotech), and 5 ng/mL recombinant human TGF-β (Prospec) (Th17 conditions). After 5 days, cells were re-stimulated with PMA and ionomycin and stained with antibodies specific for CD4, IFN-γ and IL-17 as described above.

### Preparation of Bone Marrow-Derived Dendritic cells (BMDCs), Macrophages (BMDMs) and T Cell-Depleted Splenocytes for Co-culture Studies

Bone marrow cells were flushed from the femurs and tibias of mice using syringes containing sterile 1 x PBS. Red blood cells were lysed using Lysis buffer (see above) and were resuspended in complete RPMI. For BMDC generation, bone marrow cells were cultured for 8 days in complete RPMI medium that contained 20 ng/mL GM-CSF (Prospec), which was added to the culture every 3 days. For BMDM generation, bone marrow cells were cultured for 7 days in complete RPMI medium that contained 10 ng/mL mouse M-CSF (Prospec), which was added to the culture every 2–3 days. Bulk APCs were obtained by CD4^+^ and CD8^+^ T cell depletion of splenocytes using the MACs separation system. Bulk APCs, BMDCs, and BMDMs were then stimulated overnight with 0.1 μg/mL LPS, and were washed and counted prior to co-culture with T cells.

### APC/T Cell Co-culture Assays

For BMDC co-culture studies, 2D2 T cell receptor transgenic CD4^+^ T cells were isolated from lymph nodes and spleens of 2D2 mice using the MACs separation system and were labeled with 0.5 μM CFSE. These cells were then plated together with BMDCs (ratio of 2:1) for 3 days in the presence of 10 μg/mL MOG_35-55_.

For bulk APC and BMDM co-culture studies, CD4^+^ T cells were isolated by negative selection from WT mice and plated together at various ratios with T cell-depleted splenocytes or BMDMs on anti-CD3 antibody-coated (2 μg/mL) 96 well plates. In some experiments, the medium was supplemented with 10 μM of the nitric oxide synthase 2 (NOS2)-specific inhibitor, L-N^6^-(1-Iminoethyl) lysine dihydrochloride (L-NIL, R&D Systems). After 3–5 days, cells were collected and stained for flow cytometry and supernatants were frozen for cytokine analysis by ELISA assay (BioLegend).

### Nitrite Measurements

Supernatants from macrophages stimulated overnight with 0.1 μg/mL LPS were used to measure levels of nitrite, as a measure of NO production using the Griess Reagent Kit (Life Technologies) according to methods described in the technical bulletin.

### Flow Cytometry

The following mAbs were used for flow cytometry: anti-CD4 (GK1.5), anti-IL-17 (TC11-18H10.1), anti-IFN-γ (XMG12), anti-CD3 (145-2C11), anti-CD8 (53.6.7), anti-CD11c (N418), anti-CD11b (M1/70), anti-F4/80 (BM8), anti-CD86 (GL-1), anti-CD80 (16-10A1), anti-CD40 (3/23), anti-PDL1 (10F.9G2), all from BioLegend, anti-NOS2 (CXNFT) from eBioscience and anti-MHCII (AF6-120.1) from BD Biosciences. Cell viability was assessed using eFluor450 dye (eBioscience). Viable cells were identified as the eFluor450 negative population. Data were acquired using an LSRII (BD Biosciences) cytometer and analyzed with FlowJo software (TreeStar).

### Statistical Analysis

All data are expressed as mean ± standard error of mean. Data were compared between WT and OGR1-KO mice using a student’s two-tailed t-test (if data were parametric) or a Mann-Whitney U test (if data were non-parametric). In addition, the effect of L-NIL on WT and OGR1-KO cell cultures was investigated using a one-way ANOVA and Tukey post-hoc test. A value of p<0.05 and below was considered to be significant.

## Results

### Deficiency in OGR1 Results in Attenuated EAE

**Fig 1:**
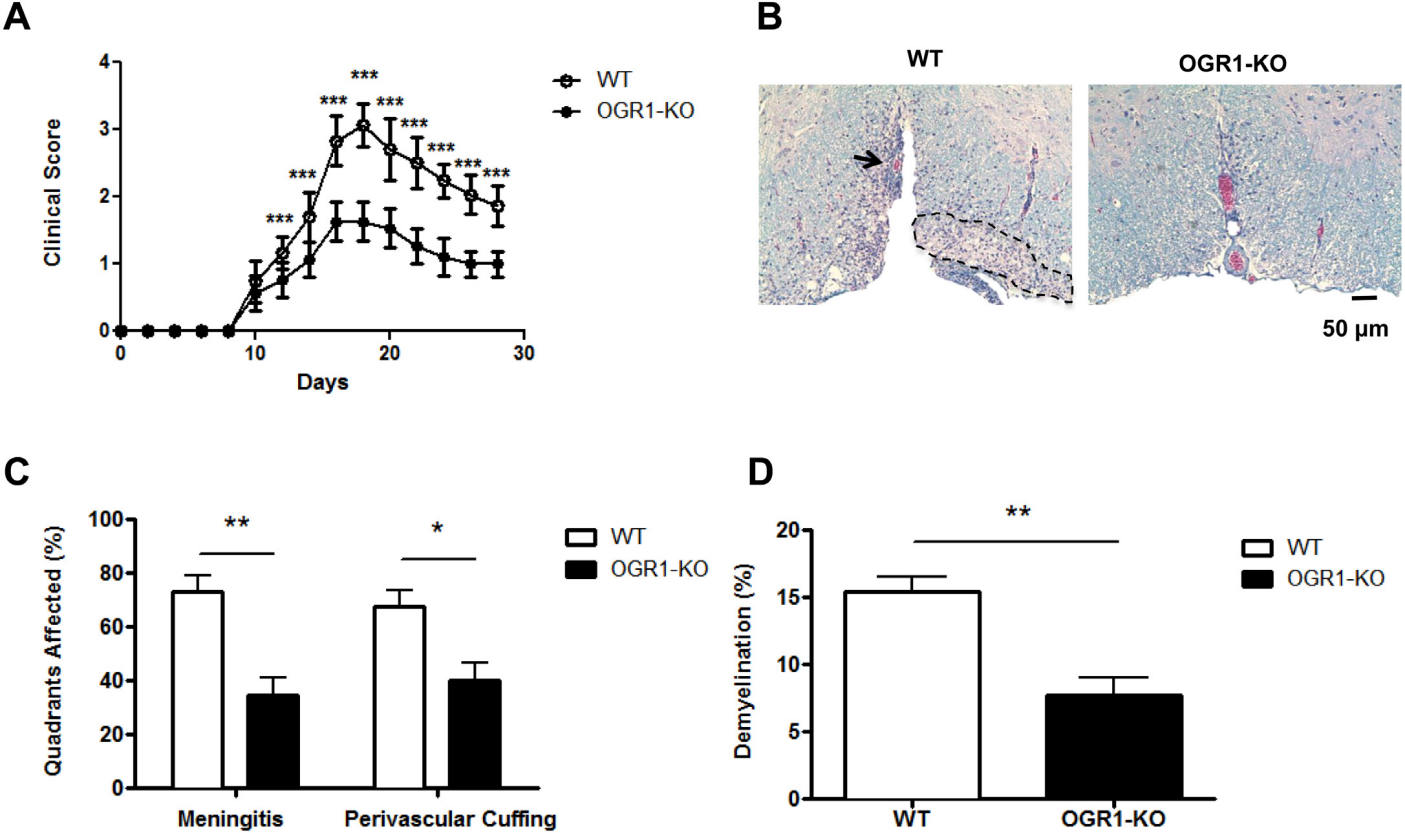
Deficiency in OGR1 results in the attenuation of EAE. (A) EAE was induced in wild type (WT) and OGR1-KO mice by injection of MOG_35-55_/CFA and pertussis toxin and mice were observed daily for clinical signs of EAE for 28 days. Data shown are means + SEM of clinical scores obtained from 3 independent experiments that each used N = 5 mice/group. (B-D) In one experiment, cross sections of WT and OGR1-KO spinal cords (N = 10 sections/mouse) were prepared from N = 8 mice/group and were stained with Luxol Fast Blue and hematoxylin and eosin. Sections were scored blindly for meningeal and perivascular inflammation and demyelination. (B) shows a representative area of a spinal cord cross section from one WT and OGR1-KO mouse. The arrow indicates perivascular infiltration and dashed outlined section indicates area of demyelination. (C) shows the percent of spinal cord quadrants that were affected by meningeal inflammation or that contained perivascular cuffs. (D) shows the percent of the spinal cord white matter that was demyelinated. In C and D, values are means + SEM of values obtained in the N = 8 mice/group. *p<0.05, **p<0.01, ***p<0.001 by two-tailed t-test.

OGR1 has recently been shown to regulate the development of tumors [17, 22] and airway inflammation [16], however its role in autoimmunity is not known. To investigate the role for OGR1 in T cell-mediated autoimmunity, we induced EAE in OGR1-KO and WT mice via immunization with MOG_35-55_/CFA and observed mice for clinical signs over a 28-day period. As seen in Fig 1A, WT mice started to show signs of disease at day 10 post-immunization and continued to progress to develop hind limb paralysis, after which they exhibited a partial recovery of hind limb function. In contrast, OGR1-KO mice showed a significantly attenuated disease course compared to WT mice at nearly all time points examined (Fig 1A and Table 1). While WT animals had a maximal clinical score of 3.1 (±0.3) with an average cumulative score of 20.8 (±2.2) after 28 days (Table 1), OGR1-KO mice did not progress beyond a clinical score of 1.7 (±0.3) and exhibited a cumulative score of 11.6 (±1.7). Histological analysis of hematoxylin/eosin- and Luxol Fast Blue-stained spinal cord sections from WT and OGR1-KO mice (Fig 1B) revealed that the reduced EAE severity in OGR1-KO mice associated with a ~50% reduction in the extent of meningeal and perivascular inflammation and demyelination in the spinal cord white matter compared to levels observed in WT mice (Fig 1C and 1D).

**Table 1:** Clinical features of disease in OGR1-KO and WT mice.

Clinical feature	WT	OGR1-KO
***Maximal score*:**		
**Experiment 1**	3.2 (0.3)	1.8 (0.3) ***
**Experiment 2**	3.1 (0.2)	1.6 (0.2) ***
**Experiment 3**	3.0 (0.4)	1.7 (0.4) ***
**Average:**	**3.1 (0.3)**	**1.7 (0.3)** ***
***Day of Onset*:**		
**Experiment 1**	10.0 (0)	10.0 (0)
**Experiment 2**	10.0 (0)	10.4 (0.9)
**Experiment 3**	10.0 (0)	10.0 (0)
**Average:**	**10.0**	**10.1**
***Cumulative Score*:**		
**Experiment 1**	21.8 (1.2)	12.4 (1.3) ***
**Experiment 2**	20.8 (2.7)	10.7 (2.0) ***
**Experiment 3**	19.9 (2.3)	11.6 (1.7) ***
**Average:**	**20.8 (2.2)**	**11.6 (1.7)** ***

Each experiment used N = 5/group. Values are means (SEM). The incidence of EAE was 100% in all three experiments that were performed.

*** p<0.0001 by Student’s t-test (two-tailed).

**Fig 2:**
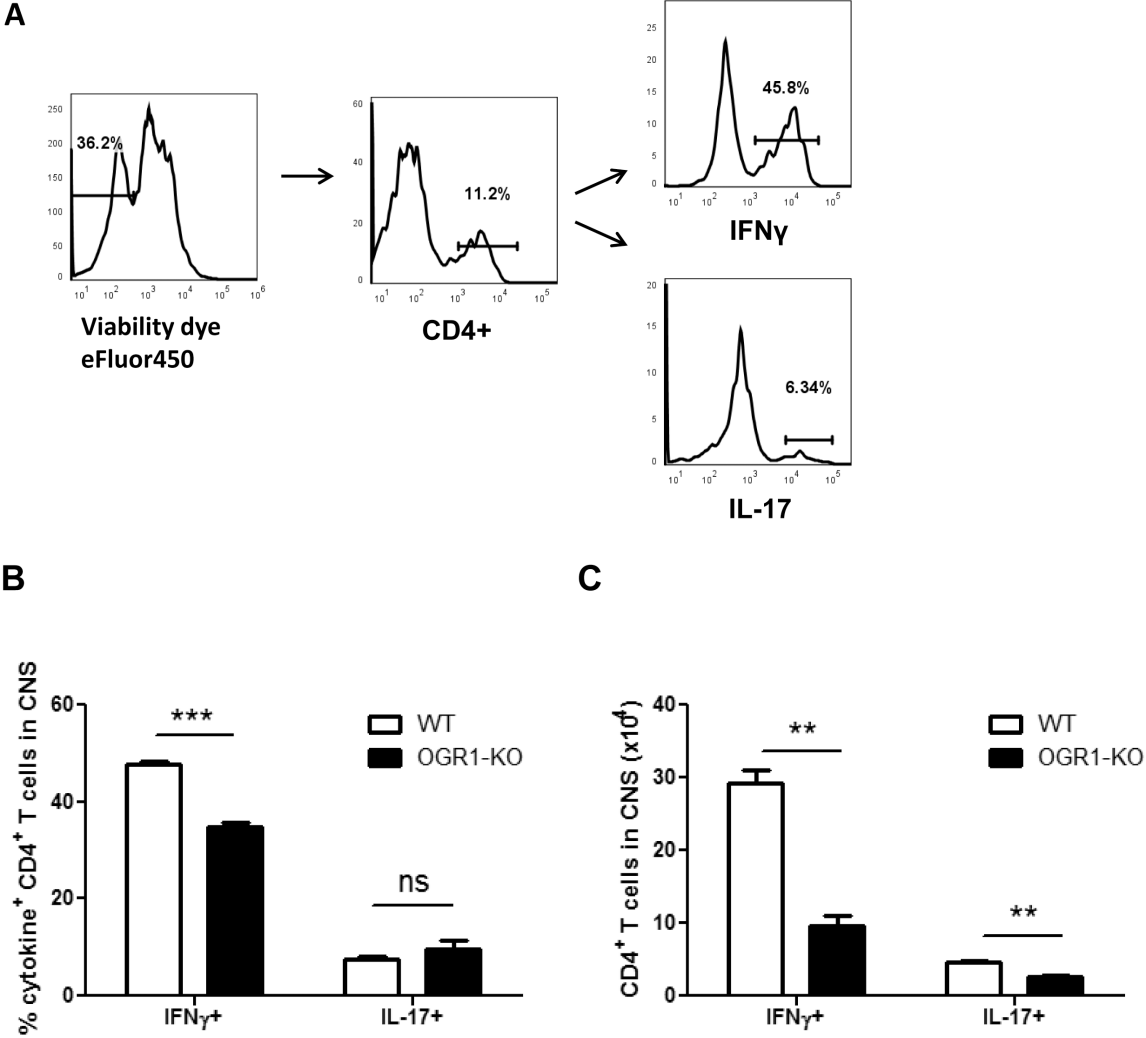
The frequency and total number of Th1 and Th17 cells were significantly reduced in OGR1-KO CNS during EAE. WT and OGR1-KO mice were immunized with MOG_35-55_/CFA and were provided pertussis toxin and at 28 days post-immunization brains and spinal cords were removed and CNS mononuclear cells were isolated by Percoll gradient centrifugation. Lymphocytes were counted, restimulated *in vitro* with PMA and ionomycin in the presence of brefeldin A, and stained with antibodies specific for CD4, IFN-γ and IL-17. (A-B) The percentages of IFN-γ^+^ and IL-17^+^ cells within the CD4^+^ gate were measured by flow cytometry and (C) the total cell number of each population was calculated. Data are means + SEM of individual mice (N = 3/group) and are representative of 3 independent experiments. **p<0.01, ***p<0.001 by two-tailed t-test. Similar studies were conducted at day 10 post-immunization with similar results.

Given that the CNS inflammatory response in EAE is initiated by Th1 and Th17 cells [1], we next measured the accumulation of Th effector cells in the CNS of OGR1-KO and WT mice during EAE. To this end, mononuclear cells were isolated from pooled spinal cord and brain tissue of WT and OGR1-KO mice, and the number and frequencies of IFN-γ- and IL-17-producing CD4^+^ T cells were evaluated by intracellular cytokine staining. As shown in Fig 2B, we observed a significantly lower frequency of IFN-γ^+^ CD4^+^ T cells in the OGR1-KO CNS compared to WT CNS. OGR1-KO mice also showed significantly reduced numbers of IFN-γ^+^ and IL-17^+^ CD4^+^ T cells in CNS tissue compared to WT animals (Fig 2C). These data coincide with the histological analysis of inflammation and suggest that OGR1 regulates the accumulation of Th1 and Th17 effectors in the CNS during EAE.

### OGR1-KO Mice Exhibit Impaired Antigen-Specific T Cell Responses During EAE

**Fig 3:**
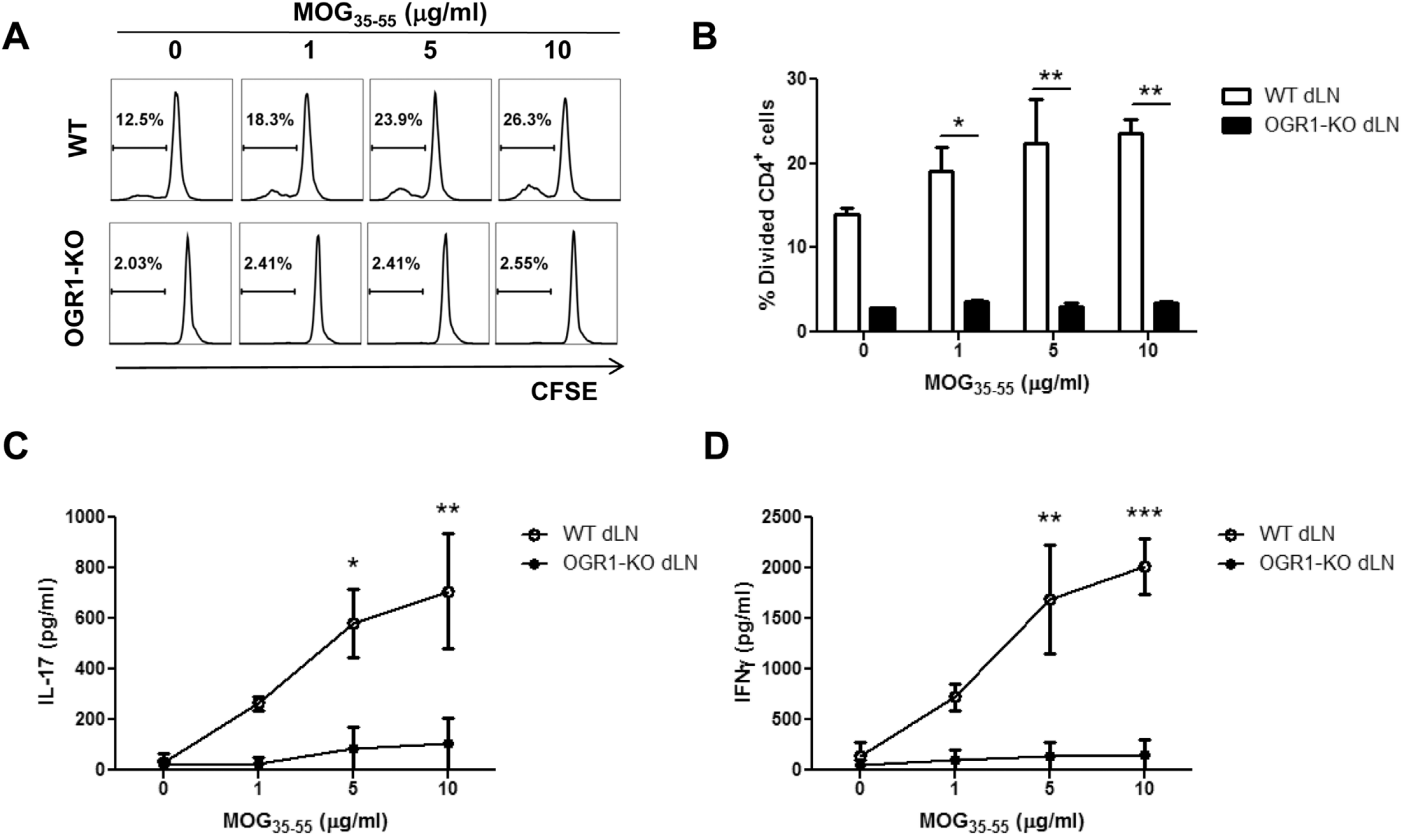
OGR1 deficiency impairs the generation of antigen-specific T cells. (A-B) Mice (N = 6/group) were immunized with MOG_35-55_/CFA and at 10 days post-immunization draining lymph nodes (dLN) were removed and were pooled together. Lymph node mononuclear cells were labeled with CFSE and cultured with MOG_35-55_ peptide (0–10 μg/mL) for 3 days. Cells were collected and stained with anti-CD4 antibody and the percentage of divided CD4^+^ cells was analyzed by CFSE dilution. (C-D) Supernatants from the cultures were used to measure the levels of IL-17 (C) or IFN-γ (D) by ELISA. Data are means + SEM of values obtained from triplicate cultures and are representative of 3 independent experiments. *p<0.05, **p<0.01, ***p<0.001 by t-test (two-tailed).

The reduced accumulation of Th1 and Th17 effectors in the CNS of OGR1-KO mice during EAE could have resulted from defects in the priming and expansion of, or cytokine production by MOG_35-55_-reactive T cells in the periphery. To address these possibilities, we measured the recall proliferation and cytokine production by antigen-specific CD4^+^ T cells in the draining lymph nodes (dLN) of WT and OGR1-KO mice upon *ex vivo* culture with MOG_35-55_. As seen in Fig 3A and 3B, dLN CD4^+^ T cells from WT mice showed a dose-dependent increase in proliferation upon stimulation with increasing concentrations of MOG_35-55_. In contrast, dLN CD4^+^ T cells from OGR1-KO mice showed very little proliferation in response to any concentration of MOG_35-55_ tested. OGR1-KO dLN cells also exhibited a lower basal division than the WT dLN cells, in wells where no exogenous MOG was added. In these wells, the T cell division observed was likely in response to antigen presenting cells (APC) presenting MOG peptide that was derived from the emulsion. The higher proliferation of the WT compared to the OGR1-KO cells at day 10 post-immunization indicates either that the WT dLNs contained a larger pool of expanded MOG-reactive T cells compared to the OGR1-KO dLNs or that OGR1-KO MOG reactive T cells had a reduced capacity than WT T cells to proliferate to antigen signals.

Following these trends in T cell proliferation, MOG_35-55_-elicited IL-17 and IFN-γ production was also significantly lowered in dLN cultures established from OGR1-KO versus WT mice (Fig 3C and 3D). Given that the cytokine production largely followed the T cell proliferation results, our data strongly suggest that there is failure in the priming or expansion of antigen-specific effector T cells in OGR1-KO animals during EAE.

### OGR1-KO CD4^+^ T Cells Show No Inherent Defects in Their Capacity to Proliferate and Differentiate

**Fig 4:**
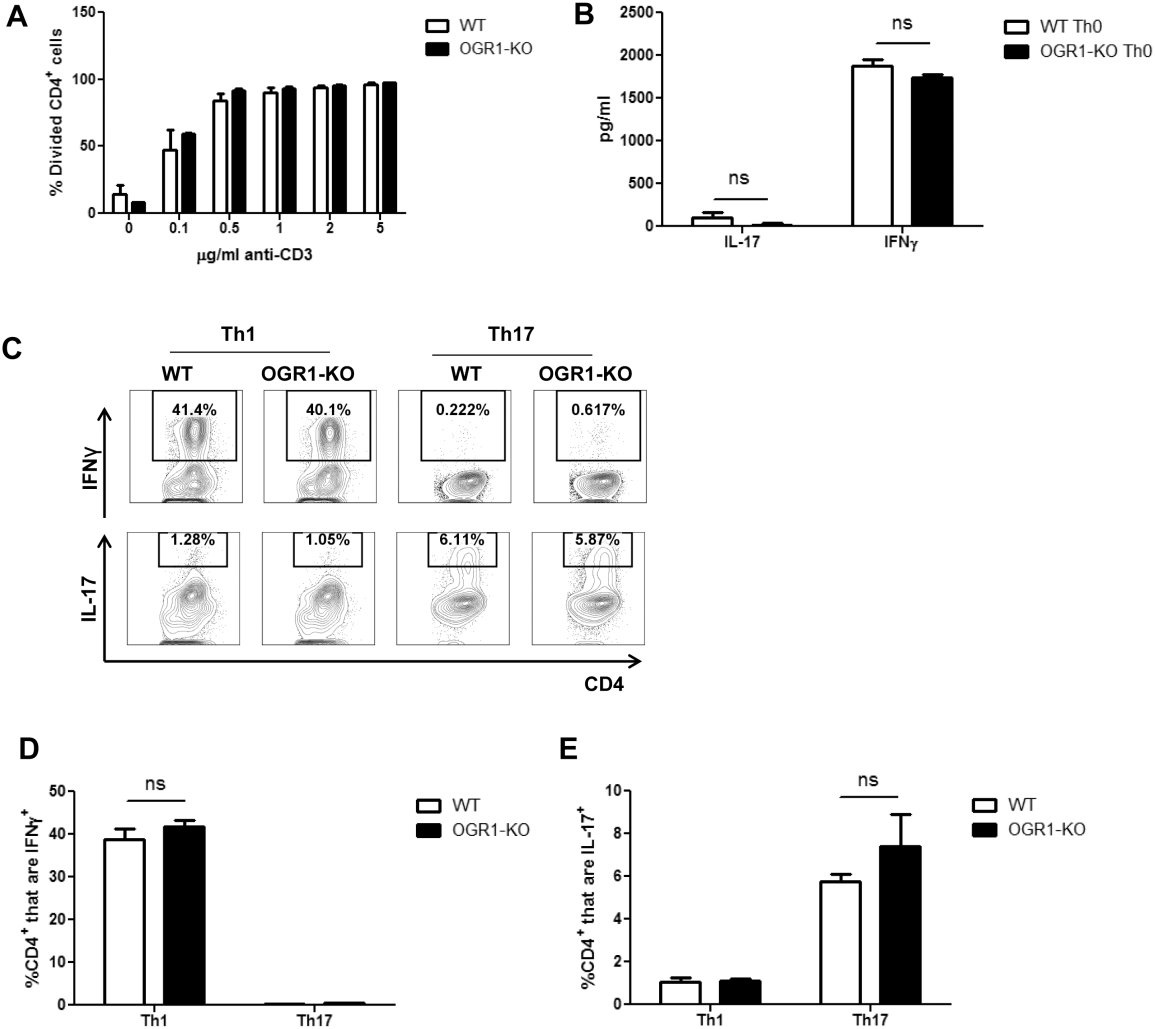
OGR1-KO T cells show no inherent defects in their capacity to proliferate and differentiate. (A) CD4^+^ T cells were isolated from WT and OGR1-KO spleen and lymph nodes (N = 4 mice/group), were pooled and labeled with CFSE and stimulated with anti-CD3 and anti-CD28 (2 μg/ml) antibodies. After 3 days of culture, cells were collected and stained with anti-CD4 antibody and cell proliferation was measured by CFSE dilution. Data are means + SEM of triplicate cultures and are representative of 4 independent experiments. (B) WT or OGR1-KO CD4^+^ T cells were cultured with soluble anti-CD28 antibody (2 μg/ml) on anti-CD3-coated (2 μg/ml) plates for 5 days and then were re-stimulated with PMA and ionomycin and IL-17 and IFN-γ were measured by ELISA. Data are means + SEM of triplicate cultures and are representative of 3 independent experiments. (C-E) CD4^+^ T cells from WT or OGR1-KO mice were stimulated with anti-CD3 and anti-CD28 (both at 2 μg/ml) under Th1 or Th17 polarizing conditions. After a 5-day incubation, cells were re-stimulated with PMA and ionomycin in the presence of brefeldin A for an additional 4 hours. Cells were then collected and stained with antibodies for CD4, IFN-γ or IL-17 and the percentage of IFN-γ^+^ and IL-17^+^ CD4^+^ cells was calculated. Results in C show representative FACs plots while data in D and E are means + SEM of triplicate cultures. Data shown are from one of three experiments that were performed. ns = not significant, by Student’s t-test (two-tailed).

The impaired generation of antigen-specific Th1 and Th17 cells observed in OGR1-KO mice could have resulted from functional defects in either the T cell compartment or the antigen-presenting cell (APC) compartment. We therefore first investigated if there were any overt differences in the T cell compartment between naïve WT and OGR1-KO mice. As seen in Tables 2 and 3, we did not observe any differences in the total number (Table 2) or percentage (Table 3) of CD3^+^, CD4^+^ or CD8^+^ T cells in the spleens or lymph nodes of naïve WT and OGR1-KO mice. We then compared the capacity of WT and OGR1-KO CD4^+^ T cells to proliferate and produce Th cytokines upon stimulation with anti-CD3 and anti-CD28 antibodies. As shown in Fig 4A, WT and OGR1-KO T cells exhibited a similar extent of proliferation as measured by CFSE dilution upon stimulation with varying concentrations of anti-CD3 and anti-CD28. In addition, WT and OGR1-KO CD4^+^ T cells secreted similar levels of IL-17 and IFN-γ upon anti-CD3 and anti-CD28 stimulation (Fig 4B). Next, to assess the ability of WT and OGR1-KO CD4^+^ T cells to differentiate into Th1 and Th17 cells, purified CD4^+^ T cells were stimulated with anti-CD3 and anti-CD28 antibodies under Th1 or Th17 polarizing conditions; however we detected no differences in the percentage of IFN-γ^+^ or IL-17^+^ CD4^+^ cells between OGR1-KO and WT cultures (Fig 4C–4E). Overall, these data indicate that OGR1 deficiency does not alter the ability of CD4^+^ T cells to proliferate or differentiate *in vitro*.

**Table 2:** Total cell numbers x 10^6^ in Naïve WT and OGR1-KO mice.

Cell Type	WT Spl	OGR1-KO Spl	WT LN	OGR1-KO LN
**CD3**^**+**^	18.72 (2.39)	17.36 (1.18)	6.90 (0.63)	8.59 (0.44)
**CD4**^**+**^	10.53 (0.60)	10.01 (0.14)	3.97 (0.19)	4.56 (0.19)
**CD8**^**+**^	7.33 (0.64)	6.57 (0.38)	2.82 (0.17)	3.11 (0.14)
**CD11c**^**+**^	0.48 (0.16)	0.49 (0.18)	0.04 (0.02)	0.03 (0.01)
**F480/CD11b**^**+**^	0.19 (0.01)	0.17 (0.04)	0.005 (0.004)	0.003 (0.001)

n = 4 mice per group. Values are means (SEM).

No significant differences were observed between WT and OGR1-KO data by two-tailed t-test.

**Table 3:** Percent of Live Cells in Naïve WT and OGR1-KO mice.

Cell Type	WT Spl	OGR1-KO Spl	WT LN	OGR1-KO LN
**CD3**^**+**^	30.40 (3.88)	31.67 (2.17)	66.87 (6.11)	69.87 (3.65)
**CD4**^**+**^	17.11 (0.98)	18.26 (0.26)	38.51 (1.88)	39.26 (0.66)
**CD8**^**+**^	11.92 (1.04)	11.98 (0.69)	27.39 (1.67)	29.69 (2.20)
**CD11c**^**+**^	0.78 (0.25)	0.90 (0.32)	0.39 (0.21)	0.24 (0.09)
**F480/CD11b**^**+**^	0.31 (0.01)	0.31 (0.07)	0.05 (0.03)	0.02 (0.01)

n = 4 mice per group. Values are means (SEM).

No significant differences were observed between WT and OGR1-KO data by two-tailed t-test.

### APC From OGR1-KO Mice Have a Reduced Capacity to Support T Cell Proliferation

**Fig 5:**
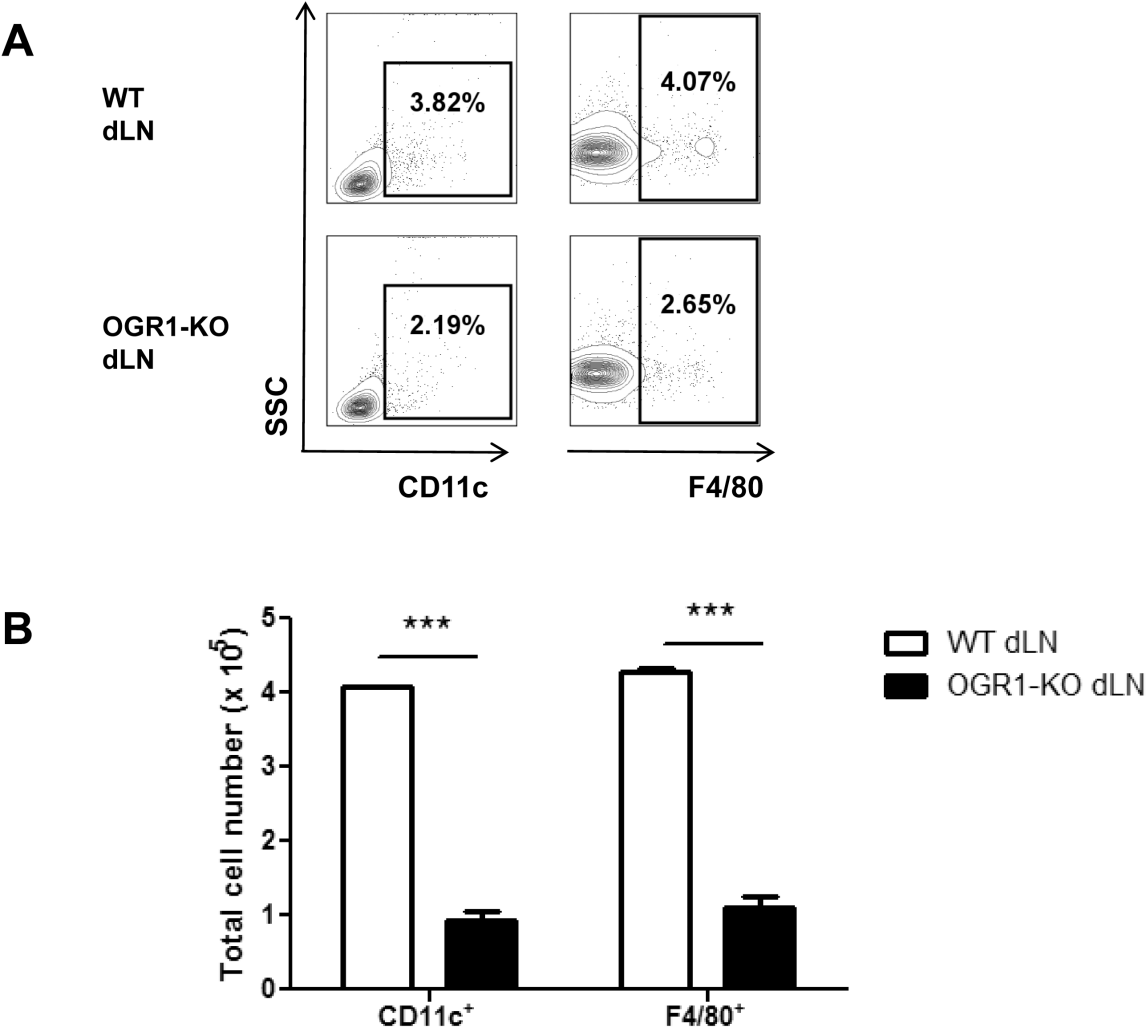
The frequency and total number of macrophages and DCs are reduced in OGR1-KO dLN after immunization. (A-B) Draining lymph nodes (dLN) were removed from WT and OGR1-KO mice at 10 days post-immunization with MOG_35-55_/CFA. The frequencies (A) and total number (B) of CD11c^+^ and F4/80^+^ cells were determined by flow cytometry. Data in A are representative flow plots, while data in B are means + SEM of values obtained from N = 6 mice/group. Data shown are representative of 2 independent experiments.

Since there appeared to be no inherent defects in the T cell compartment in OGR1-KO mice, we investigated whether the APC compartment was altered in these mice. First, we examined macrophage and dendritic cell populations in the spleen and LNs of naive WT and OGR1-KO mice; however we detected no aberrations in either the total cell number or frequencies of these APC populations in these immune organs in OGR1-KO mice (Tables 2 and 3). Next, to determine if there were any differences in these APC populations during EAE, we also quantified CD11c^+^ and F4/80^+^ cells in the dLNs at 10 days post-immunization. Under these conditions, we detected a 40% reduction in the frequency (Fig 5A) and a 75% reduction in the total number (Fig 5B) of CD11c^+^ and F4/80^+^ cells in OGR1-KO compared to WT animals. This finding suggested a possible defect in the maturation or recruitment of APC to dLNs during EAE.

**Fig 6:**
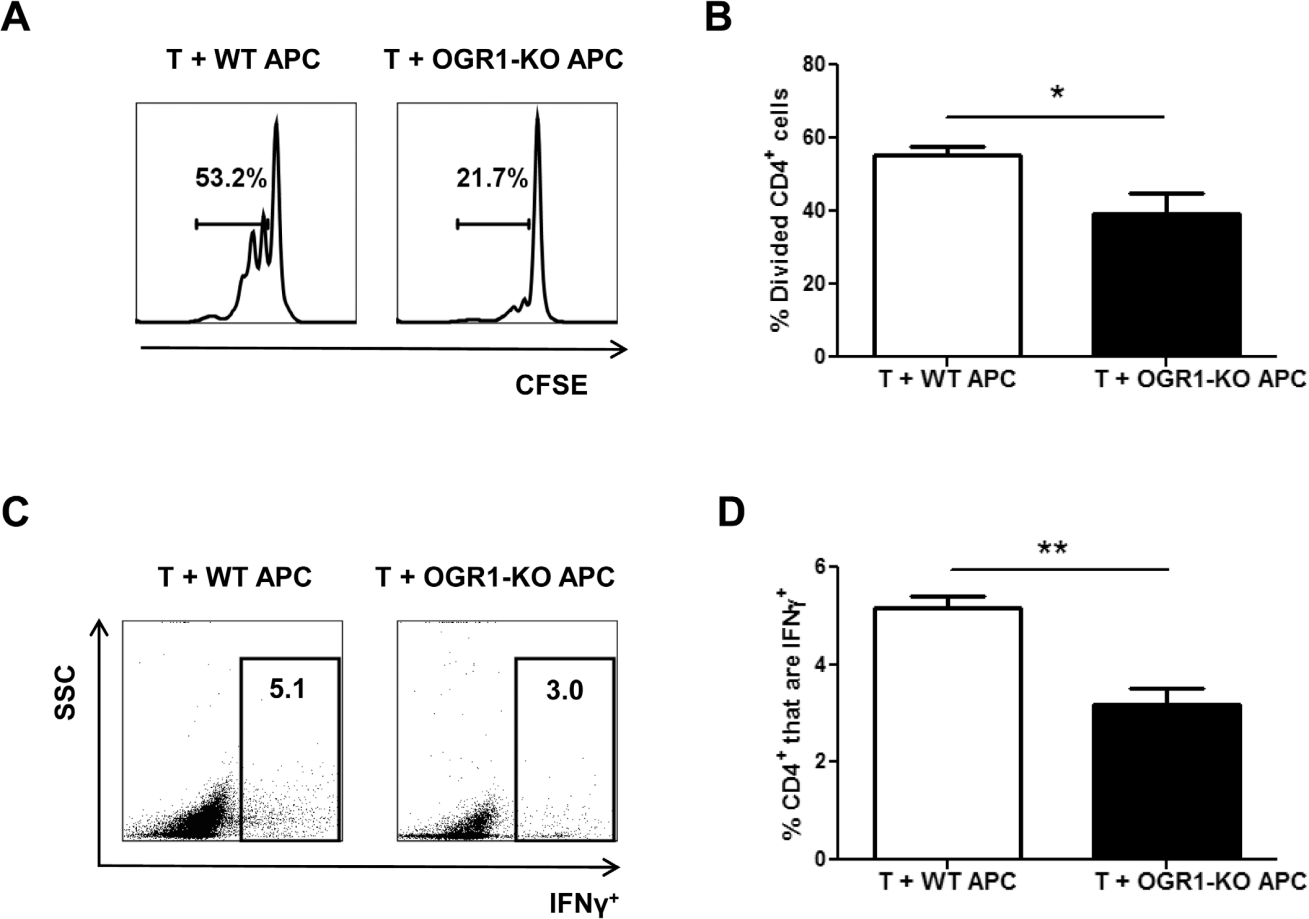
APC from OGR1-KO mice have a decreased ability to support T cell proliferation and cytokine production. Splenocytes were isolated from WT and OGR1-KO mice and T cells were depleted by MACs separation as described in the Methods. T cell-depleted splenocytes (APC) were stimulated with LPS overnight, washed and co-cultured (1:1 ratio) with CFSE-labeled WT CD4^+^ T cells that were plated onto anti-CD3 antibody-coated plates. The percentage of divided CD4^+^ cells (A-B) was examined after 3 days of culture. Data shown are from 4 individual experiments. (C-D) Cells were stimulated with PMA, ionomycin in the presence of brefeldin A for an additional 4 hours and intracellular staining for IFN-γ was performed. (C) shows representative FACs plots of IFN-γ expression versus side scatter of CD4^+^ cells and (D) shows the means + SEM of values obtained from 3 individual experiments. *p<0.05, **p<0.01 by t-test (two-tailed).

We next investigated the ability of WT and OGR1-KO APCs to prime CD4^+^ T cells by co-culturing an equal number of T cell-depleted splenocytes (as a source of bulk APC) from WT or OGR1-KO mice with WT T cell responders in the presence of anti-CD3. We observed that CD4^+^ T cells co-cultured with OGR1-KO APCs proliferated to a much lesser extent compared to CD4^+^ T cells co-cultured with WT APCs (Fig 6A and 6B). In addition, we detected a lower frequency of Th1 effector cells in the co-cultures that contained OGR1-KO APC versus those that contained WT APC (Fig 6C and 6D). We also measured frequencies of IL-17^+^ cells in these co-cultures, but observed very low percentages of these cells in both groups (data not shown). These data indicate that deficiency in OGR1 is associated with a lowered frequency of DCs and macrophages in dLNs during EAE and a reduced capacity of APC to support Th cell expansion.

### OGR1-KO macrophages have a greater ability than WT macrophages to inhibit the proliferation and cytokine production by CD4^+^ T cells

Next we explored which type of APC was responsible for the attenuated disease observed in KO animals. In the MOG peptide-based model of EAE, dendritic cells have been implicated as having the major role in the priming of myelin-reactive T cells [23]. We therefore compared the ability of OGR1-KO and WT bone marrow-derived dendritic cells (BMDC) to prime CD4^+^ T cells. As shown in S1A Fig, we observed no defects in the maturation of BMDCs in response to GM-CSF, nor in the ability of these cells to express MHC Class II or co-stimulatory molecules after overnight stimulation with LPS (S1B Fig). Similarly, when BMDCs from OGR1-KO mice were co-cultured with 2D2 T cell receptor transgenic T cells in the presence of MOG_35-55_ antigen, we observed that OGR1-KO BMDCs were just as effective as WT BMDCs at supporting CD4^+^ T cell proliferation (S1C and S1D Fig). Together these findings suggest that dendritic cell differentiation and function were not compromised in OGR1-KO mice.

**Fig 7:**
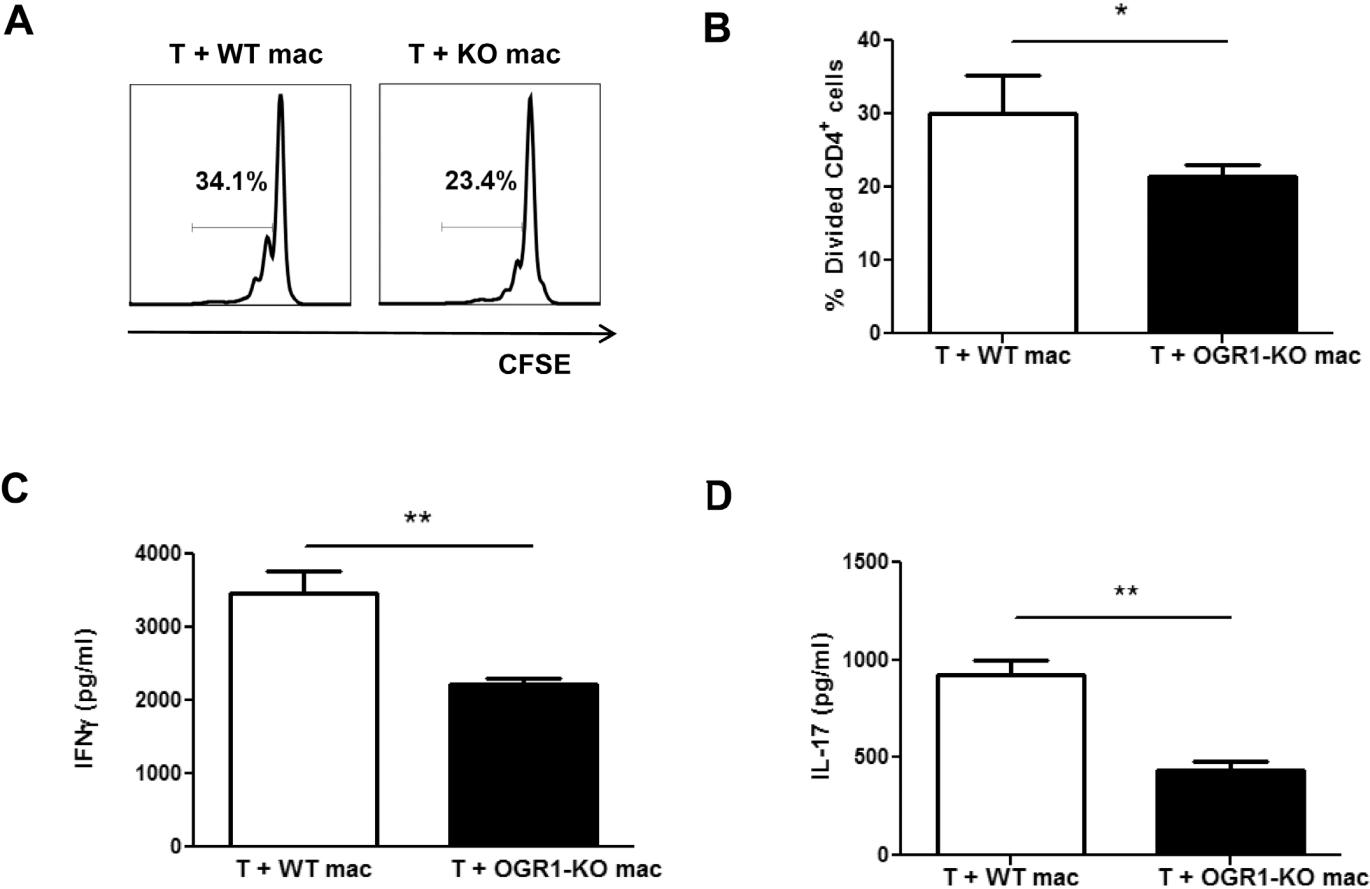
OGR1-KO macrophages have a greater ability than WT macrophages to inhibit the proliferation and cytokine production by CD4^+^ T cells. CFSE-labeled WT CD4^+^ T cells were plated onto anti-CD3 antibody-coated plates and co-cultured with either WT or OGR1-KO macrophages (mac) that had been pre-stimulated with 0.1 μg/mL LPS. The percentage of divided CD4^+^ cells (A-B) was examined after a 3-day incubation period. (A) shows representative data while (B) shows means + SEM of values obtained from 6 individual experiments. *p< 0.05 by Student’s t-test (two-tailed). (C-D) WT CD4^+^ T cells were co-cultured with either WT or OGR1-KO macrophages, at a ratio of 1:0.2, for 5 days (C) or 4 days (D) and supernatants were used to measure the levels of IFN-γor IL-17 by ELISA. Data are means + SEM of triplicate cultures and are representative of 3 independent experiments. *p<0.05, **p<0.01 Student’s t-test (two-tailed).

Since macrophages also function as APC and play important roles in EAE development [24–26], we next examined if macrophage function was altered by OGR1 deficiency. Similar to findings for BMDCs, we did not observe any differences in the yields or maturation status of WT and OGR1-KO bone marrow-derived macrophages (BMDM) (S2A and S2B Fig). Yet consistent to our findings of bulk splenocytes, we observed that CD4^+^ T cells exhibited impaired proliferation (Fig 7A and 7B) and Th cytokine production (Fig 7C and 7D) when co-cultured with OGR1-KO compared to WT macrophages. We also noted that there was a tendency for a higher frequency of dead (positive for viability dye) CD4^+^ T cells in the co-cultures that contained OGR1-KO versus WT BMDMs (18.7 + 0.4 in OGR1-KO versus 8.7 + 3.7 in WT, Mann-Whitney U test, P = 0.055). Collectively, these data suggest that OGR1-KO macrophages are more potent than WT macrophages at suppressing the proliferation of Th1 and Th17 effector cells.

### Altered NO Production Accounts for the Differential Ability of OGR1-KO and WT Macrophages to Support T Cell Proliferation

To further explore what feature was responsible for the differential effect of OGR1-KO and WT macrophages on T cell proliferation, we compared the expression of co-stimulatory and co-inhibitory molecules and the production of cytokines between OGR1-KO and WT BMDMs. As shown in S2B Fig, WT and OGR1-KO macrophages expressed similar levels of CD86, CD80, CD40, MHCII and PD-L1 after overnight stimulation with LPS. Similarly, we detected no significant differences in the production of pro-inflammatory (IL-12p40, IL-12p70, IL-6) or anti-inflammatory (IL-10 and TGFβ) cytokines between OGR1-KO or WT macrophages after LPS stimulation (S2C Fig). We also investigated the productions of IL-1β and IL-23 in culture supernatants from LPS-elicited macrophages, but did not detect these cytokines above background levels (data not shown).

**Fig 8:**
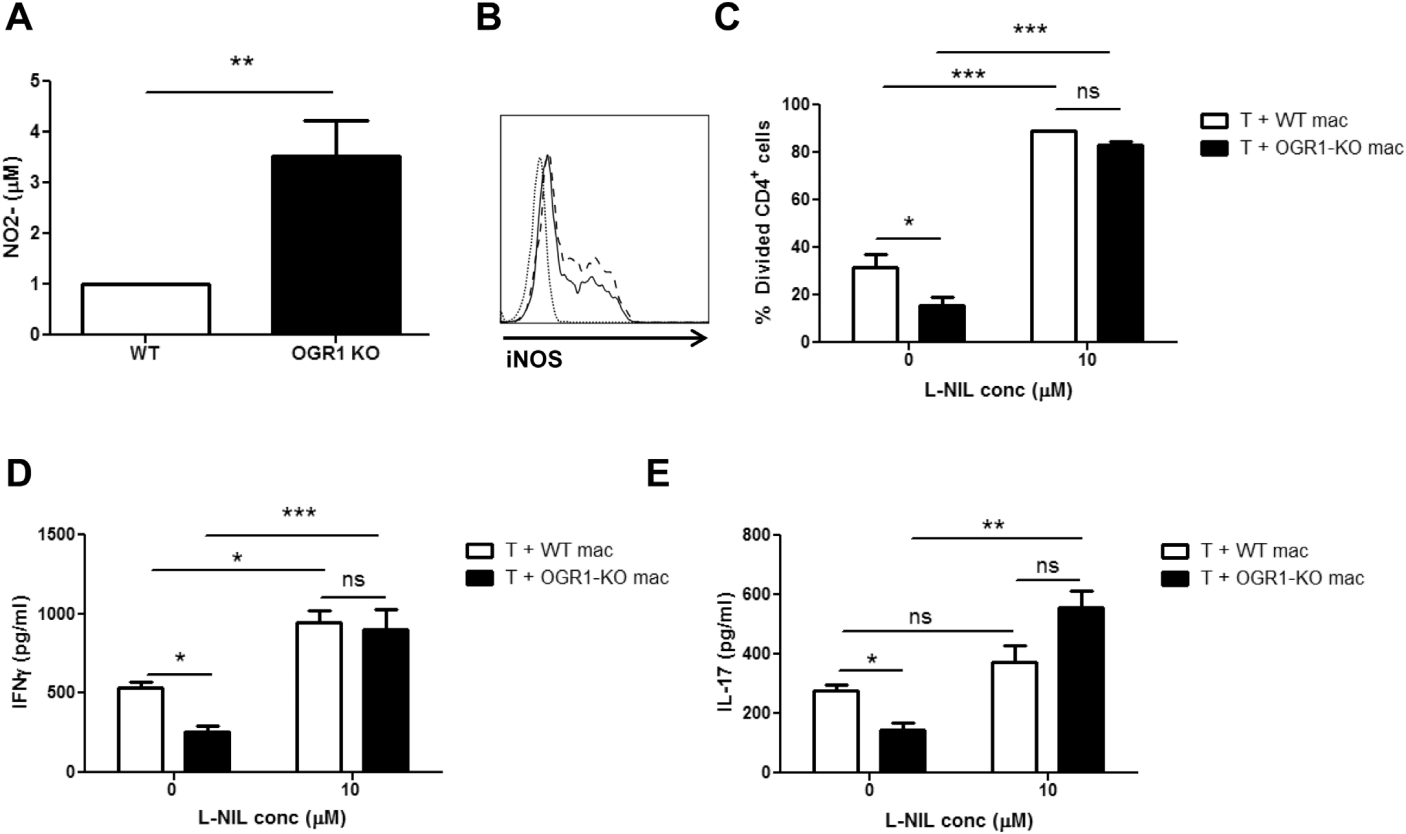
Differential NO Production Accounts for the Differential Ability of OGR1-KO and WT Macrophages to Support T Cell Proliferation. (A) WT and OGR1-KO macrophages were stimulated with 0.1 μg/mL LPS overnight and supernatants were collected and analyzed for nitrite concentration using the Griess assay. Data are means + SEM of values obtained from N = 5 mice/group. (B) The expression of iNOS was examined by flow cytometry in WT (solid line) and OGR1-KO (dashed line) macrophages after stimulation with 0.1 μg/mL LPS overnight. Histograms of cells positive for iNOS, within the CD11b^+^F4/80^+^ gate, are shown. Staining with the isotype control antibody (dotted line) is also shown. This panel is representative of 3 experiments that were performed on N = 3 mice/group. (C-E) WT CD4^+^ T cells and either WT or OGR1-KO macrophages (mac) were co-cultured in the presence or absence of 10 μM L-NIL. (C) The percent of divided CD4^+^ cells was determined by staining CFSE-labeled T cells in the co-culture with anti-CD4 antibody after a 3-day incubation. (D-E) Supernatants were collected and analyzed for IFN-γ and IL-17 by ELISA. Values in C-E are means + SEM of results from triplicate cultures and are representative of 3 independent experiments that were performed. *p<0.05, **p<0.01, ***p<0.001 by Student’s t-test (two-tailed) or one-way ANOVA.

Past studies have shown that macrophages can suppress T cell proliferation through nitric oxide (NO) secretion [27–31]. In addition, NO-induced immunosuppression and T cell killing have been identified as a mechanism of how macrophages attenuate EAE induction [32, 33]. We therefore examined whether there were differences in the secretion of NO production by WT and OGR1-KO macrophages. Interestingly, after stimulation with LPS, OGR1-KO macrophages secreted NO at ~ 4-fold higher levels than WT macrophages, as detected by nitrite assay (Fig 8A). The increased NO secretion by OGR1-KO relative to WT macrophages correlated with an increased expression of iNOS at the protein level (Fig 8B). To determine if this increased NO production was the factor that accounted for the reduced T cell proliferation in the macrophage/T cell co-cultures, we performed co-culture experiments in the presence and absence of the NOS2-specific inhibitor, L-NIL. Consistent with our previous observations, culture with OGR1-KO macrophages resulted in reduced levels of T cell proliferation and IFN-γ and IL-17 secretion compared to culture with WT macrophages (Fig 8C–8E). Importantly, treatment with 10 μM L-NIL significantly increased the proliferation and the cytokine production by CD4^+^ T cells, particularly in the co-cultures that contained the OGR1-KO macrophages (Fig 8C–8E). Together, these data confirm an immunosuppressive role for macrophage-derived NO on T cell proliferation and further suggest that the higher macrophage NO secretion contributed to the impaired T cell responses in OGR1 mice.

## Discussion

Although considerable insight has been gained into the pathobiological and immunological mechanisms involved in MS and the animal model EAE, the etiology of MS has proven elusive and new targets for therapy are continually being evaluated. Recent studies have implicated a pro-inflammatory role for the proton-sensing GPCR, OGR1, in a variety of diseases including allergy and cancer; however whether this molecule has a similar role in autoimmunity had not been examined. Here we explored the role of OGR1 in the development of EAE and observed that OGR1-KO mice exhibited a striking attenuation of CNS inflammation during EAE that associated with a reduced expansion of MOG_35-55_-reactive Th1 and Th17 cells in the periphery and a reduced accumulation of Th1 and Th17 effector cells in the CNS. In addition, our studies elucidated that impaired T cell responses in OGR1-KO mice associated with a reduction in the numbers of DCs and macrophages in dLNs during EAE and higher production of NO by macrophages. Taken together, our studies suggest that OGR1 plays an important role in controlling T cell expansion in the early stages of EAE.

Our finding that OGR1-KO mice exhibited a defective expansion of Th cells during EAE is consistent with previous observations of reduced adaptive immune responses in OGR1-KO mice in a syngeneic prostate tumor model [17] and in the OVA-induced sensitization/challenge model of asthma [16]. In the prostate cancer model, it was observed the spleen mononuclear cells that were harvested from OGR1-KO-tumor-bearing mice exhibited impaired productions of both pro- and anti-inflammatory cytokines compared to WT counterparts upon re-challenge with tumor cells *ex vivo* [17]. Similarly, in the asthma model, it was observed that OGR1-KO mice exhibited reduced airway inflammation than WT mice that associated with lowered Th2 cytokine production and decreases in plasma OVA-specific IgE [16]. Further consistent with a pro-inflammatory role for OGR1, a recent study reported that OGR1-KO mice also develop less severe colitic inflammation against enteric antigens in the IL-10^-/-^ model of irritable bowel disease [13]. Collectively, these results suggest that OGR1-KO mice are less able to mount T cell adaptive immune responses, regardless of the nature of the inciting antigen (i.e., ovalbumin, MOG, tumor cells, microbiota) or the Th bias of the disease model.

Regarding the cellular mechanism of the reduced T cell expansion in OGR1-KO mice, our data ruled out a role for OGR1 in the T cell compartment, since OGR1-KO mice did not exhibit any defects in the frequencies of T cells in peripheral lymphoid organs, nor in the ability of these cells to proliferate and differentiate upon anti-CD3 and anti-CD28 stimulation. Instead, our studies identified a number of defects in the APC compartment that associated with the impaired Th1 and Th17 responses in OGR1-KO mice: (1) OGR1-KO mice exhibited a reduced frequency and number of dendritic cells in inflamed lymph nodes during EAE and (2) OGR1-KO macrophages exhibited a higher production of NO in response to innate stimuli.

Regarding APC numbers, we had observed that OGR1-KO mice exhibited a lowered frequency of macrophages and DCs in the dLNs compared to WT mice at 10 days post-immunization. Differences in the frequencies of these cells were not apparent in the lymph nodes of naive WT versus OGR1-KO mice. We also did not observe a difference in the ability of OGR1-KO DCs or macrophages to differentiate from bone marrow precursors in culture. Thus, we speculate that the reduced frequency of these DCs and macrophages in the OGR1-KO dLNs during EAE is either (1) a secondary consequence of the OGR1-KO lymph nodes being less inflamed (less chemokines produced to attract DCs and macrophages), or (2) is due to a migratory defect in APC populations in the OGR1-KO mouse. In regard to the latter, past studies have provided evidence for a migratory defect of macrophages and DCs in the OGR1-KO mouse. Li and colleagues reported that OGR1-KO mice exhibited a defect in the recruitment of macrophage precursors to the peritoneum after thioglycollate injection [14]. Furthermore, Aoki et al. reported in an asthma model that OVA-pulsed DCs from OGR1-KO mice were less able than WT DCs to migrate to the peribronchial lymph nodes to cause airway inflammation [16]. This group further showed that this defective migration of the OGR1-KO DCs correlated with lowered expression of CCR7 by OGR1-KO DCs [16]. Whether, this mechanism also accounts for the lowered numbers of DCs or macrophages observed in OGR1-KO dLNs during EAE is unclear and will be examined in future studies.

In addition to the reduction in the frequency of DCs, we further observed that OGR1-KO macrophages produced higher levels of NO than WT macrophages upon LPS stimulation. This observation is consistent with previous findings in the prostate cancer model, where it was reported that lowered tumor engraftment in OGR1-KO mice correlated with higher tumor expression of NOS2 and higher macrophage tumoricidal activity [17]. Given that high NOS2 expression is a defining feature of pro-inflammatory or M1 macrophages [34], Yan and colleagues had also concluded that OGR1 deficiency resulted in a shift from an M2 towards an M1 macrophage phenotype [17]. However, we profiled the expression levels of a number of markers and cytokines known to be enriched in M1 macrophages (IL-12p40, IL-12p70, IL-6, MHC Class II, CD80, CD86) [34], yet observed no differences between WT and OGR1-KO macrophages. Thus, we conclude that OGR1 activity does not necessarily shift macrophage profile from M1 to M2, but negatively regulates one aspect of macrophage function, arginine metabolism and the production of NO. Thus, OGR1 joins a growing list of factors (i.e., IFN-γ, TNF, IL-1β, IL-33, osmolarity, hypoxia) that regulate NO production by macrophages [35].

Our finding that treatment with L-NIL abrogated the suppressive effect of OGR1-KO macrophages on T cell proliferation also pointed to the higher NO production as a cause of the T cell immunosuppression. Notably, we observed that T cell proliferation was also enhanced by L-NIL treatment in the co-cultures that contained the WT macrophages; albeit to a more limited extent than OGR1-KO cells. This latter observation is consistent with previous findings of inhibition of T cell expansion by macrophage-derived NO in WT mice [24–27] and reports that blockade of NOS2 activity or genetic deficiency in NOS2 worsens Th effector function during EAE [32, 33, 36], whereas treatment with NO donors is protective in this disease [37]. Future studies will determine whether there is a link between the dysregulated NO production by OGR1-KO macrophages and the defective T cell expansion in OGR1-KO mice during EAE.

Though our study did not elucidate the mechanism of how NO inhibited T cell proliferation, past studies have observed that macrophage-derived NO can lead to reductions in the tyrosine phosphorylation of Jak3/STAT5 downstream of the IL-2R [38, 39]. It was further shown that this effect of NO on Jak3 was reversed by guanylate cyclase inhibitors implicating the classic pathway of guanylate cyclase activation and cGMP generation in NO-mediated immunosuppression [38]. In addition, studies in other cell types have demonstrated that NO can interfere with tyrosine phosphorylation in cells through cGMP-independent mechanisms including formation of peroxynitrate and tyrosine nitration [35]. Thus, the enhanced NO production by OGR1-KO macrophages may have inhibited T cell proliferation through a variety of mechanisms.

A previous study reported that pH decreases to 6.6 in the interstitial fluid in the inflamed spinal cord during EAE [5]. Though we did not measure pH within the inflamed lymph nodes, we noted modest decreases in pH in our *in vitro* cultures of dLNs in buffered culture media (from 7.7 in the wells without added peptide to 7.2–7.3 in wells containing peptide). These decreases were in a range known to activate OGR1 [9]. These findings warrant future studies to determine how decreases in pH activate OGR1 to modulate NOS2 expression in macrophages. In addition, past reports have demonstrated that activation of OGR1 through extracellular acidification leads to increases in intracellular calcium through phospholipase C/IP3 signaling in various cell types studied and triggers an accumulation of cAMP in human vascular smooth muscle cells [40]. Thus, studies investigating the signaling pathways that operate downstream of OGR1 in macrophages will also help to further understand the molecular mechanisms by which NOS2 expression is regulated.

In addition to inhibiting T cell proliferation, NO is reported to promote Th1 polarization of CD4^+^ T cells [41–43]. In this regard, it has been shown that treatment with chemical NO donors promotes Th1 differentiation by upregulating IL-12 receptor β2 expression [43] and inhibits Th17 differentiation through tyrosine nitration on RORγt [41] or through upregulation of the aryl hydrocarbon receptor in T cells [42]. Moreover, a number of studies have reported that macrophage-derived NO can induce T cell apoptosis [31, 44]. While we did note a tendency for higher T cell death in the co-cultures that contained the OGR1-KO macrophages, we did not observe any indications in our *in vivo* or *in vitro* studies that OGR1-deficiency or L-NIL treatment altered the ratio of Th1 to Th17 cells. The discrepancy between our data and past findings likely relates to relatively modest changes in NO production (4-fold increase) that occurred with OGR1-deficiency. In support of this notion, it is reported that concentrations of chemical NO donors required to inhibit T cell proliferation (10–100 μM), are lower than the levels that induce T cell apoptosis or inhibit Th17 differentiation (>100 μM) [42, 44]. Overall, our findings suggest that OGR1 modulates NO production by macrophages within a modest range to selectively impact T cell proliferation.

In addition to mediating immunosuppression and T cell death, NO has a number of functions that may be detrimental to EAE and MS [45–47]. For example, treatment with NO donors is reported to increase the permeability of the blood brain barrier in rats [48], which may increase the migration of immune cells in the CNS. Furthermore, *in vitro* studies have demonstrated that NO is a major cytotoxic factor for oligodendrocytes [49, 50] and neurons [51]. Reactive nitrogen species and reactive oxygen species also damage mitochondria within neurons, which is thought to be the major pathological factor underlying MS progression [52]. Our finding that OGR1-KO mice were still susceptible to EAE despite the profound suppression of T cell expansion was somewhat surprising and hints that OGR1 may modulate more distal events in EAE development such as immune cell recruitment to the CNS or inflammation within the EAE lesion. Future experiments using an adoptive T cell transfer approach will help to further distinguish the role of OGR1 on T cell priming from more distal mechanisms in EAE development.

In conclusion, our data demonstrate that OGR1 deficiency results in attenuated autoimmune inflammation due to a defect in the expansion of myelin-reactive T cells in the periphery. These results suggest that manipulating OGR1 activity may be a novel way of modulating T cell responses in autoimmunity and other T cell-mediated diseases.

## Supporting Information

S1 Fig BMDCs from OGR1-KO mice show no developmental or functional defects. (A) BMDCs were grown from the bone marrow of WT and OGR1-KO mice in the presence of GM-CSF. After 8 days, cells were stimulated overnight with LPS. Shown is a representative plot of CD11c^+^ antibody staining of these cells. The number of cells collected from plates was also similar in the 5 experiments that were performed. (B) Flow analysis was also performed to measure the cell surface expression of CD86, CD80, CD40, MHC Class II, and PD-L1 on WT (solid line) and OGR1-KO (dashed line) CD11c^+^ DCs. Shown are representative FACs plots from one experiment of 4 that were performed. (C-D) CD4^+^ T cells were isolated from spleen and LNs from 2D2 mice, labeled with CFSE and co-cultured with either WT or OGR1-KO BMDCs at 2:1 ratio in the presence or absence of MOG_35-55_ peptide. After 3 days, cells were collected, stained with anti-CD4 antibody and the percentage of divided cells was analyzed by flow cytometry by measuring CFSE dilution on the CD4^+^ population.
(TIF)

S2 Fig BMDMs from OGR1-KO mice show no developmental or cytokine defects. (A) BMDMs were grown from the bone marrow of WT and OGR1-KO mice in the presence of M-CSF and were stimulated overnight with 0.1 μg/mL LPS and then stained with antibodies to CD11b, F4/80, CD86, CD80, CD40, MHC Class II and PDL1. (A) Shows representative FACs plots of CD11b^+^ and F480^+^ staining in BMDM cultures. (B) Representative histograms of the expression of co-stimulatory markers on WT (solid line) and OGR1-KO (dashed line) CD11b^+^F4/80^+^ macrophages. Isotype controls are shown as dotted lines. (C) Cytokines were measured in either WT or OGR1-KO macrophage supernatants at 24 h post-LPS stimulation by ELISA assay. Data are means + SEM of values obtained from 8 cultures. ns = not significant by t-test (two-tailed).
(TIF)
