# Supplementary figures and images for: OGR1/GPR68 Modulates the Severity of Experimental Autoimmune Encephalomyelitis and Regulates Nitric Oxide Production by Macrophages
Source: PLoS One. 2016 Feb 1;11(2):e0148439. doi: 10.1371/journal.pone.0148439 (PMC4735495; doi:10.1371/journal.pone.0148439)

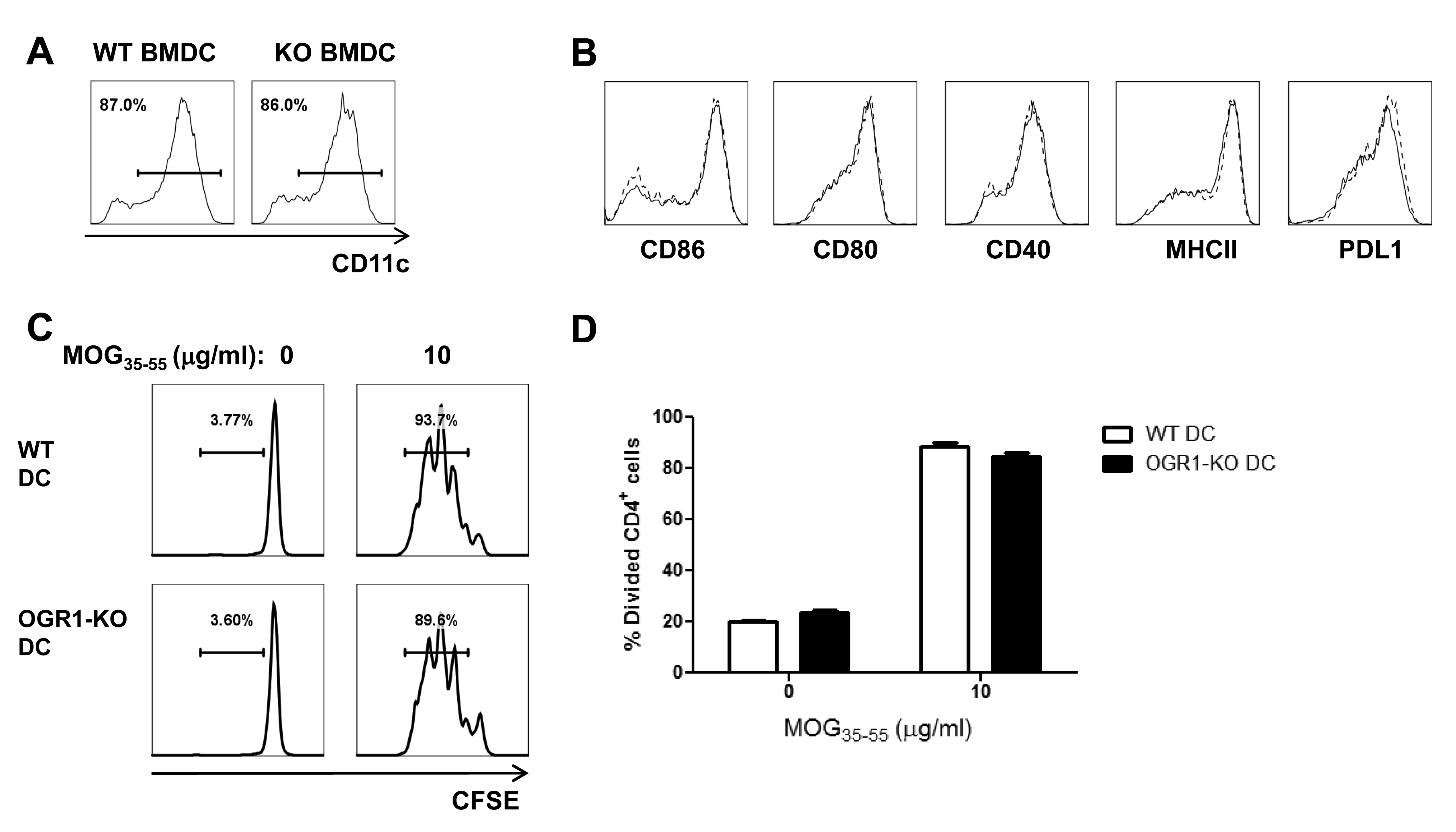

Supplement: S1 Fig — (A) BMDCs were grown from the bone marrow of WT and OGR1-KO mice in the presence of GM-CSF. After 8 days, cells were stimulated overnight with LPS. Shown is a representative plot of CD11c+ antibody staining of these cells. The number of cells collected from plates was also similar in the 5 experiments that were performed. (B) Flow analysis was also performed to measure the cell surface expression of CD86, CD80, CD40, MHC Class II, and PD-L1 on WT (solid line) and OGR1-KO (dashed line) CD11c+ DCs. Shown are representative FACs plots from one experiment of 4 that were performed. (C-D) CD4+ T cells were isolated from spleen and LNs from 2D2 mice, labeled with CFSE and co-cultured with either WT or OGR1-KO BMDCs at 2:1 ratio in the presence or absence of MOG35-55 peptide. After 3 days, cells were collected, stained with anti-CD4 antibody and the percentage of divided cells was analyzed by flow cytometry by measuring CFSE dilution on the CD4+ population. (TIF) [file pone.0148439.s001.tif]

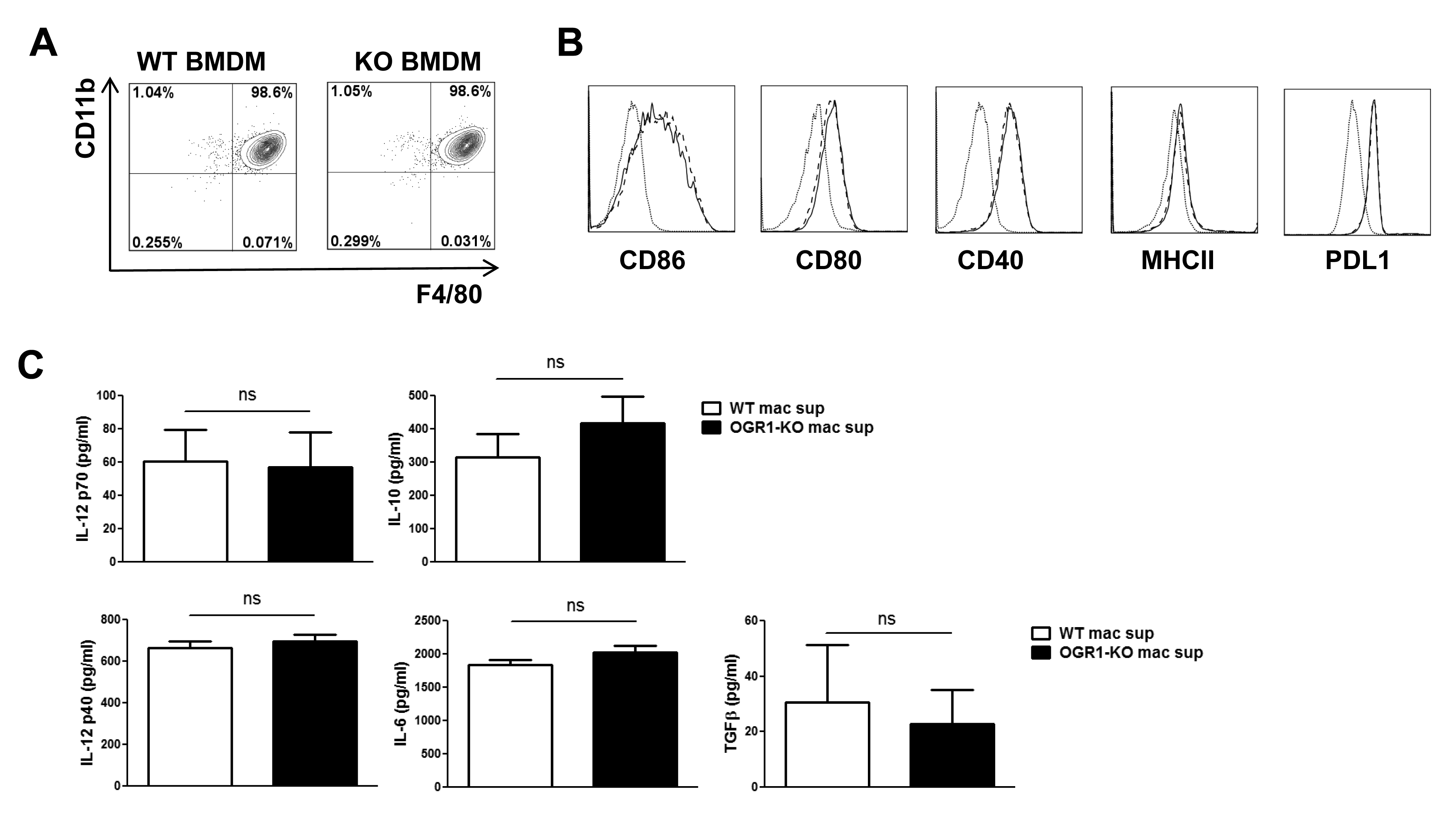

Supplement: S2 Fig — (A) BMDMs were grown from the bone marrow of WT and OGR1-KO mice in the presence of M-CSF and were stimulated overnight with 0.1 μg/mL LPS and then stained with antibodies to CD11b, F4/80, CD86, CD80, CD40, MHC Class II and PDL1. (A) Shows representative FACs plots of CD11b+ and F480+ staining in BMDM cultures. (B) Representative histograms of the expression of co-stimulatory markers on WT (solid line) and OGR1-KO (dashed line) CD11b+F4/80+ macrophages. Isotype controls are shown as dotted lines. (C) Cytokines were measured in either WT or OGR1-KO macrophage supernatants at 24 h post-LPS stimulation by ELISA assay. Data are means + SEM of values obtained from 8 cultures. ns = not significant by t-test (two-tailed). (TIF) [file pone.0148439.s002.tif]
